# Effect of Composition on the Crystallization, Water Absorption, and Biodegradation of Poly(*ε*-caprolactam*-co-ε*-caprolactone) Copolymers

**DOI:** 10.3390/polym12112488

**Published:** 2020-10-27

**Authors:** Yuanyuan Dou, Xinyu Mu, Yuting Chen, Zhenbo Ning, Zhihua Gan, Ni Jiang

**Affiliations:** State Key Laboratory of Organic-Inorganic Composites, Beijing Laboratory of Biomaterials, College of Life Science and Technology, Beijing University of Chemical Technology, Beijing 100029, China; 2018201134@mail.buct.edu.cn (Y.D.); 2019201175@mail.buct.edu.cn (X.M.); 2016201147@mail.buct.edu.cn (Y.C.); zbning@mail.buct.edu.cn (Z.N.); zhgan@mail.buct.edu.cn (Z.G.)

**Keywords:** poly(ester amide), composition, crystallization, biodegradation

## Abstract

Poly(ester amide)s have aroused extensive research interest due to the combination of the degradability of polyester and the higher mechanical properties of polyamide. In this work, a series of poly(*ε*-caprolactam*-co-ε*-caprolactone) (P(CLA*-co-*CLO)) copolymers with different compositions were synthesized by anionic copolymerization. The structure, crystallization behavior, water absorption, and biodegradation behavior of these copolymers were investigated by means of nuclear magnetic resonance (NMR), Fourier transform infrared (FTIR), differential scanning calorimetry (DSC), wide-angle X-ray diffraction (WAXD), and polarized optical micrographs (POM). The results indicated that the composition of P(CLA*-co-*CLO) copolymers can be adjusted by the molar feed ratio. The PCL blocks decreased the crystallization rate of PA6 blocks but had little effect on the melting behavior of PA6, while the crystallized PA6 acted as a heterogeneous nucleating agent and greatly improved the crystallization rate of PCL. Moreover, the introduction of PCL blocks greatly reduced the water absorption of P(CLA*-co-*CLO) copolymers and endow them a certain degree of degradability.

## 1. Introduction

Aliphatic polyesters are a type of biodegradable polymer that have attracted great interest in the fields of biomedicine, agriculture, and packaging [1]. Poly(*ε*-caprolactone) (PCL) is a widely used commercial aliphatic polyester with good biocompatibility, degradability, and crystallinity. However, the poor mechanical strength of PCL limits its application [2]. Polyamide (PA) is currently one of the engineering plastics with the largest consumption and the widest application range. Strong hydrogen bonds formed between amide groups impart excellent thermal and mechanical properties, but on the other hand, they result in high moisture absorption and the low-temperature impact strength of PA [3,4]. Poly(ester amide)s are very promising materials combining the degradation ability of polyesters and the higher thermal and mechanical properties of PA [5,6].

For poly(*ε*-caprolactam*-co-ε*-caprolactone) (P(CLA*-co-*CLO)), the current researches mainly focus on the synthesis and structure characterizations of copolymers. Many synthetic methods have been reported, such as anionic copolymerization, interfacial copolymerization, hydrolytic ring-opening polymerization, and polycondensation [7,8,9,10,11,12,13,14]. Regardless of the synthesis method and feeding sequence, the final products obtained are almost random or multiblock copolymers so far. Even though the diblock and triblock poly(ester amide)s have been reported by Deshayes [9], the block sequence was imperfect due to the one-pot method.

Besides, the effect of chemical structure on the thermal property and biodegradation behavior of P(CLA*-co-*CLO) has been referred in some literature. Goodman and Vachon [7] synthesized a series of P(CLA*-co-*CLO) copolymers with variant monomer ratios; they found that the melting temperature (*T*_m_) and glass transition temperature (*T*_g_) were related to the chemical compositions. Deshayes and According to the DSC and WAXS results, Michell et al. [5,9,15,16,17] found that only the CLA sequence of P(CLA*-co-*CLO) copolymers could crystallize except for C_55_-*ran*-A_45_, and the crystallization temperature (*T*_c_) shifts were affected by microstructure and the dilution effect due to the CLO interruptions. Sanchez et al. [11] reported that only in the case of P(CLA_19_-*co*-CLO_81_), both CLA and CLO sequences could crystallize, but only a single melting peak. Though the above works found both CLA and CLO sequence could crystallize through DSC melting curves, no extensive studies were carried out. Besides, Zeng et al. [12] found that only the CLA sequence or the CLO sequence crystallized when the CLO content was lower or higher than 70 mol% as revealed by WAXD results. All the above works only focused the effect of CLO sequence on the crystallization of CLA sequence but ignored the effect of amorphous CLA sequence or the first formed CLA crystals on the crystallization of CLO sequence. Regarding the degradation of P(CLA*-co-*CLO) copolymers, Gonsalves et al. [8,18] concluded that they could be degraded by microorganisms and hydrolysis, and the mechanism is mainly the decomposition of ester groups into carboxyl and hydroxyl groups. Deshayes et al. [5,14,16] compared the hydrolytic and enzymatic degradation, and confirmed that the biodegradability was enhanced by the increasing content of CLO. SEM micrographs showed that in a wide range of CLO content, the surface of the degraded sample showed erosion, voids and holes. Strangely, however, the degradation rate of some CLO-*ran*-CLA copolymers were found to be faster than that of PCL homopolymer, and even total degradation was achieved for some copolymers. This phenomenon was attributed to the amorphous CLO sequences.

In this work, a series of P(CLA*-co-*CLO) were successfully synthesized with caprolactam sodium as the catalyst and diisocyanate C20P as the activator, and the chemical structures was characterized by ^1^H NMR, ^13^C NMR, and FTIR. Moreover, the effect of one component on the crystallization behavior of the other one was investigated by DSC, WAXD and POM. The water absorption of P(CLA*-co-*CLO) copolymers with different composition and crystallinity was studied in detail. Also, the biodegradation behavior of copolymers was investigated by weight loss measurement and compared with the results of other work.

## 2. Experiments

### 2.1. Materials

The *ε*-caprolactam (CLA) purchased from Tokyo Chemical Industry Co., Ltd. (Shanghai, China) was recrystallized in cyclohexane three times. After drying in a vacuum oven, it was stored under argon atmosphere. *ε*-caprolactone (CLO) was purchased from Alfa Aesar (Shanghai, China) Chemicals Co., Ltd., which was purified by distilling under reduced pressure and stored in argon atmosphere before use. Amano Lipase PS from Burkholderiacepacia and 1,1,1,3,3,3-Hexafluoro-2-propanol (HFIP, 99.5%) were purchased from Sigma-Aldrich. Sodium hydroxide (NaOH), Diisocyanate C20P, deuterochloroform (CDCl_3_, purity ≥ 99.99%), and methanol were used as obtained. Commercial grade PCL (*M*_n_ = 80 kg/mol) was purchased from Sigma-Aldrich (Shanghai, China). 

### 2.2. Synthesis of P(CLA-co-CLO) Copolymers 

A certain amount of CLA and NaOH (1 mol% with respect to comonomer) was added in 50 mL ampoules tube under argon atmosphere and vacuumed for 30 min at room temperature. Then, the tube was put into oil bath at 100 °C to remove water under vacuum for 20 min. After purging with argon, a certain volume of CLO and C20P (0.5 mol% with respect to comonomer) was added into the reaction tube under argon atmosphere. Then, the reaction was carried out at 180 °C for 5 h. After quenching the reaction tube under water flow, the product was dissolved in HFIP and precipitated with an excess of methanol for three times. The final product was dried in a vacuum oven at 40 °C for 24 h before use. C_x_*-co-*A_y_ was used to denote the copolymers, where x and y related to the molar feed ratio of CLO and CLA. For example, C_75_*-co-*A_25_ means the molar feed ratio of CLO and CLA is 75:25. It should be noted that the amount of activator used to synthesize C_75_*-co-*A_25_ is twice that of C_75_*-co-*A_25_-a and the other samples, which is 1.0 mol% of comonomer, since we found that the composition of C_75_-*co*-A_25_-a is similar to that of C_90_-*co*-A_10_ according to the ^1^H NMR and FTIR results. In order to provide more information about the impact of composition on performance, C_75_-*co*-A_25_ was selected for the performance testing. The synthetic scheme is as following: 

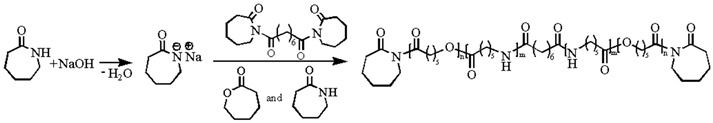



### 2.3. Water Absorption Test

The compression-molding films used for water absorption test were 1.3 × 1.3 cm^2^ with 0.1 mm thickness, which were naturally cooled at room temperature from molten state or first isothermally crystallized at 160 °C and then at 40 °C. The initial film mass was recorded as *W*_0_, and immersed in deionized water at 40 °C for 48 h. After cooling to room temperature, the sample was wiped with lens cleaning paper and weighed again, the mass of which was recorded as *W*. The water absorption was calculated as follows:

$$Water\;absorption\;\left(\%\right)\;=\frac{W}{W-W_{0}}\times \;100\%$$

### 2.4. Enzymatic Degradation

The samples for degradation were compression-molding films with 1.3 × 1.3 × 0.01 cm^3^, which were naturally cooled at room temperature from molten state. After weighing, the films were put into 3 mL phosphate buffer solution with an Amano Lipase PS concentration of 0.5 mg/mL. The samples were incubated at 37 °C, 150 rpm for different times. Besides, the degradation liquid was replaced every six hours and washed with deionized water. Then, the films were weighed after freeze-dried for 24 h. The values of weight loss were calculated from the average values of weight loss of three films. The mean errors were less than 5%. The weight loss was calculated as follows:

$$Weight\;loss\;\left(\%\right)\;=\;\frac{W_{0}-W_{t}}{W_{0}}\;\times \;100\%$$

where *W*_0_ and *W_t_* are the initial mass and the remaining mass after enzymatic degradation for different *t* of dry films. 

### 2.5. Characterizations

The chemical structure of P(CLA*-co-*CLO) copolymers was analyzed by nuclear magnetic resonance (NMR) (Bruker Advance AVANCE III, 400 MHz). The mixture of CDCl_3_/HFIP (volume ratio is 50:50) was used as a solvent for ^1^H NMR and ^13^C NMR measurement. 

Fourier transform infrared (FTIR) analysis was conducted by Perkin-Elmer Spectrum 100, the scanning wavenumber range of is 500–4000 cm^−1^ with 8 scans. The samples used for FTIR measurement were compression-molding films.

Differential Scanning Calorimetry (DSC) Q2000 (TA instruments) was used to characterize the thermal properties. About 5–8 mg samples were kept at 250 °C for 3 min to release the thermal history, and were then cooled to 10 °C at 40 °C /min and kept at 10 °C for 2 min. After that, the samples were heated to 250 °C at 20 °C/min. The samples with the same thermal history as that used for WAXD experiment were cooled to 0 °C at 40 °C/min and then heated to 250 °C at 10 °C/min to give the *T*_m_.

The wide-angle X-ray diffraction (WAXD) experiments were conducted by a D/Max 2500 VB2+/PC diffractometer (Rigaku, Tokyo, Japan) with Cu Ka radiation. The scanning rate was 5°/min and scanning range was 5–40°. The compression-molded films were firstly isothermally crystallized at 160 °C for 2 h, and then 40 °C for 2 h. 

The crystal morphology was analyzed by polarized optical microscopy (POM) (Axioskop40 A Pol, Nikon, Tokyo, Japan). The P(CLA*-co-*CLO) copolymers were dissolved in HFIP with a concentration of 40 mg/mL at 35 °C for 2 h, except for PA6 solution with a concentration of 20 mg/mL. The thin film was obtained through casting on coverslips. The film was melted at 245 °C for 2 min, then crystallized at 40 °C for 3 h or firstly crystallized at 160 °C for 1 h and then at 40 °C for 3 h. 

After melting at 245 °C for 2 min, the in-situ spherulitic morphology was observed in Linkam stage directly at 40 °C or firstly crystallized at 160 °C for 1 h, and then cooled to 40 °C at 50 °C/min for crystallization.

## 3. Results and Discussion

### 3.1. Structure Characterization

**Figure 1:**
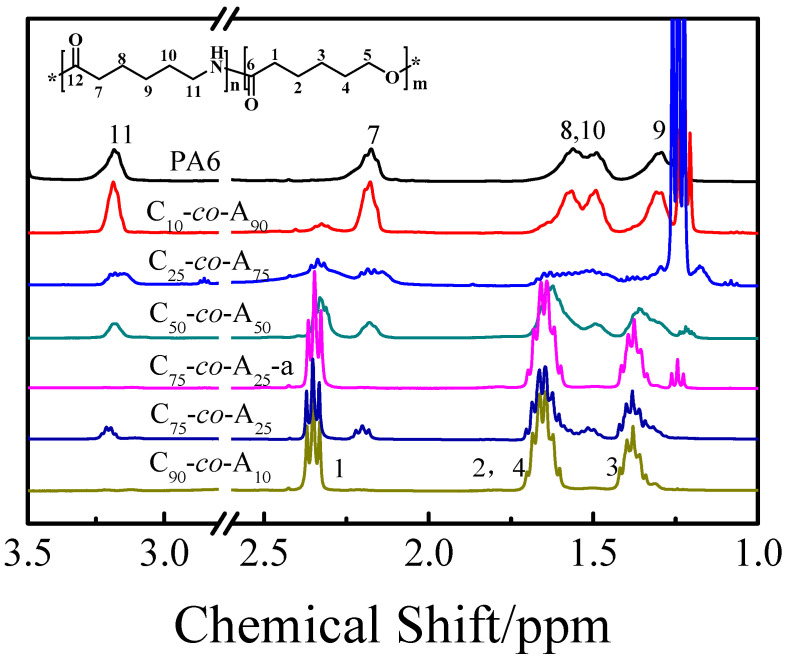
^1^H nuclear magnetic resonance (NMR) spectra of P(CLA-*co*-CLO) copolymers. (solvent: 50/50 (volume ratio) CDCl_3_/HFIP, 400MHz).

P(CLA-*co*-CLO) copolymers with different compositions were synthesized through anionic copolymerization with caprolactam sodium as catalyst and diisocyanate C20P as activator. The chemical structure and composition of the P(CLA-*co*-CLO) copolymers were characterized by ^1^H NMR and ^13^C NMR. Table 1 lists the monomer molar feed ratio and the experimental value calculated based on the ^1^H NMR results. From the ^1^H NMR spectra shown in Figure 1, it was found that the major resonances corresponding to the protons of various monomer units appeared for P(CLA-*co*-CLO) copolymers except for C_75_*-co-*A_25_-a and C_90_*-co-*A_10_. The peaks at 1.29 and 1.29–1.46 ppm are ascribed to the γ-protons of CLA and CLO units, respectively. The broad peaks around 1.41~1.73 ppm are attributed to the β- and δ-protons of CLO and CLA, the integration area of which is twice the γ-proton peak. The characteristic peaks around 2.18 and 2.35 ppm are attributed to the α-protons of CLA and CLO units, respectively. The molar ratios of the products shown in Table 1 is calculated according to the integration ratio of these two characteristic peaks. It was found that the calculated contents of CLA units in all copolymers are lower than those designed ones. This result may be caused by the low solubility of PA6 in the mixed solvents during the ^1^H NMR characterization. Although there is a certain gap between the feed and calculated molar ratio, the content of CLO units in the product increases with the increase of the feed molar ratio of CLO and CLA monomers. The ^1^H NMR characterization showed that the poly(ester amide) structure with different compositions was successfully obtained.

**Figure 2:**
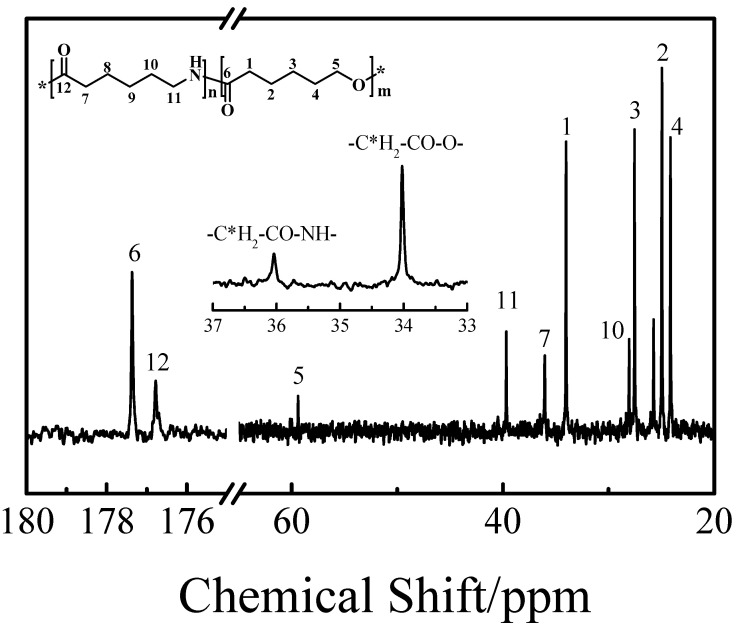
^13^C NMR spectrum of C_50_*-co-*A_50_. The insert is the expansion of the 33–37 ppm chemical shift. (solvent: 50/50 (volume ratio) CDCl_3_/HFIP, 400 MHz).

Figure 2 presents the ^13^C NMR of C_50_*-co-*A_50_. It was found that all the carbon chemical shifts correspond to the desired chemical structure. In order to clarify the sequence distribution of C_50_-*co*-A_50_, the expansion of 33–37 ppm chemical shift range of the ^13^C NMR spectrum is shown in Figure 2. No peak splitting is observed for the α-methylene peaks of the two comonomers, indicating that the randomness of the synthesized copolymer is low. 

**Figure 3:**
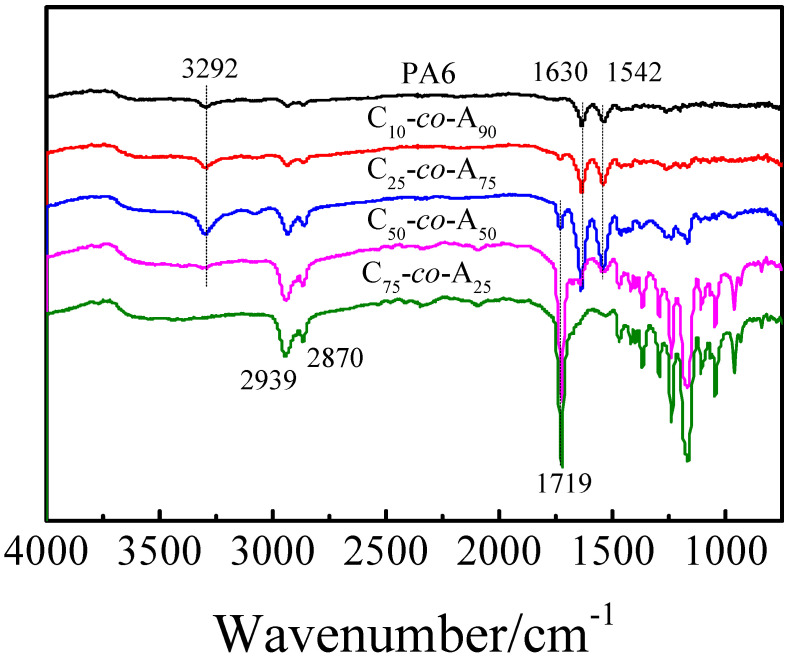
FTIR spectra of P(CLA-*co*-CLO) copolymers.

The chemical structure of all samples was further characterized by FTIR spectroscopy, and the spectra were shown in Figure 3. A wide absorption peak at 3292 cm^−1^ and two strong characteristic peaks at 1630 and 1542 cm^−1^ are assigned to the N–H stretching vibration, C=O stretching vibration (amide I band) and C–N stretching and C(O)–N–H bending vibration (amide II band) of PA6, respectively. Meanwhile, the sharp peak at 1719 cm^−1^ is assigned to the C=O stretching vibration of PCL. The appearance of the peak at 1719 cm^−1^ demonstrated the incorporation of PCL. From Figure 3 it can be seen the relative peak intensities of the peak at 1719 cm^−1^ and the peak at 1630 cm^−1^ increase with the feed molar ratio of CLO and CLA increase, indicating that the copolymer composition is adjustable, which is consistent with the ^1^H NMR results. Thus, it was concluded that a series of P(CLA-*co*-CLO) copolymers with different compositions were obtained through anionic polymerization.

### 3.2. Crystallization Behavior

**Figure 4:**
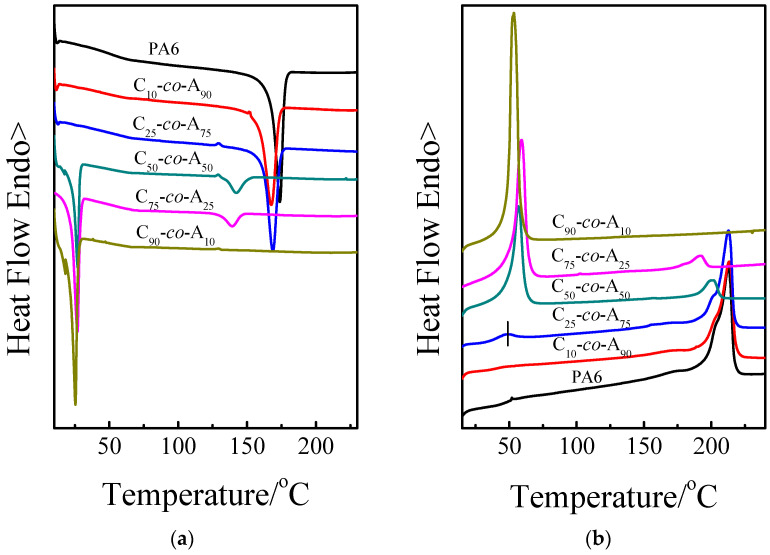
(**a**) The DSC cooling and (**b**) the second heating scans of P(CLA-*co*-CLO) copolymers. (The cooling and heating rate were 40 and 20 °C/min respectively.).

Figure 4 shows the cooling and the second heating DSC curves of P(CLA-*co*-CLO) copolymers. Two crystallization peaks were found for the C_50_*-co-*A_50_ and C_75_*-co-*A_25_, indicating that both PA6 and PCL blocks can crystallize during the cooling process. For the crystallization of PCL blocks, it was found that the crystallization temperatures (*T*_cc_) of C_50_*-co-*A_50_ and C_75_*-co-*A_25_ are even higher than that of C_90_*-co-*A_15_, indicating that the crystallized PA6 blocks act as heterogeneous nucleating agents and lead to the faster crystallization rate of PCL blocks. However, as for the crystallization of PA6 blocks, it was found that the *T*_cc_ decreases with a decrease content of PA6. On one hand, the actual content of PA6 is much lower when the CLA and CLO feed molar ratio is lower than 75:25, which may result in lower sequence length of PA6. On the other hand, the PCL blocks interrupt the crystallization of PA6 blocks, which is proved by the results of PA6 isothermal crystallization (For simplicity, the relative results are not shown here).

As for the melting behavior, the P(CLA-*co*-CLO) copolymers with double crystallization peaks show double melting peaks. For the PCL blocks, the melting temperatures (*T*_m_) of C_50_*-co-*A_50_ and C_75_*-co-*A_25_ are even higher than that of C_90_*-co-*A_10_, which may result from the higher *T*_cc_ induced by the PA6 crystals. Moreover, a lower *T*_m_ (49.4 °C) with a lower enthalpy value appears for C_25_*-co-*A_75_, indicating the crystallization process during a brief isothermal at 10 °C. For PA6 blocks, with the introduction of lower content of PCL, the *T*_m_ of PA6 remains unchanged though the *T*_cc_ decreased compared with that of neat PA6, [19] indicating that the flexible PCL is beneficial for increasing the lamellar thickness of PA6. When the content of PCL further increased, the *T*_m_ greatly decreased, which is ascribed to the lower sequence length of PA6. 

**Figure 5:**
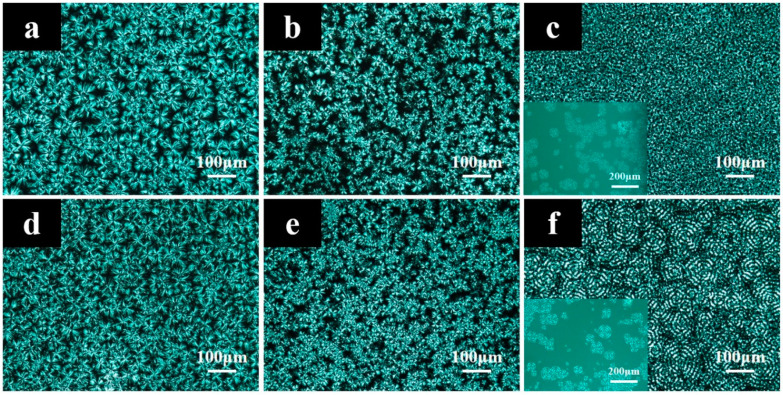
Polarized optical microscopy images of C_90_-*co*-A_10_ (**a**,**d**), C_75_-*co*-A_25_ (**b**,**e**) and C_50_-*co*-A_50_ (**c**,**f**) after isothermal crystallization directly at 40 °C (**a**–**c**) and firstly at 160 °C and then at 40 °C (**d**–**f**).

Some research work [5,11,12] also involves the crystallization behavior of P(CLA-*co*-CLO) copolymers, but because it is difficult for PCL blocks to crystallize in most case for their synthesized copolymers, the influence of PA6 blocks on the crystallization behavior of PCL blocks has never been discussed. Therefore, this influence was further investigated in this work, and the spherulitic morphologies of P(CLA-*co*-CLO) copolymers after different thermal history are shown in Figure 5. For C_90_*-co-*A_10_, the spherulitic morphology of PCL after first isothermal crystallization at 160 °C and then 40 °C is similar to that of direct isothermal crystallization at 40 °C, this is because PA6 blocks keep their amorphous state after being crystallized at 160 °C which is proven by the following WAXD results. When the content of PA6 is higher enough to crystallize, it was found that the size of PCL spherulites with the PA6 firstly crystallized is smaller than that directly crystallized at lower temperature, suggesting that the crystallized PA6 acts as heterogeneous nucleating agent, which is consistent with the DSC results. The formation of small spherulites is beneficial for PCL to maintain its high toughness. Besides, the ring-banded spherulites were observed for C_50_*-co-*A_50_, which depend on the chemical composition and the crystallization of PA6 [20,21].

**Figure 6:**
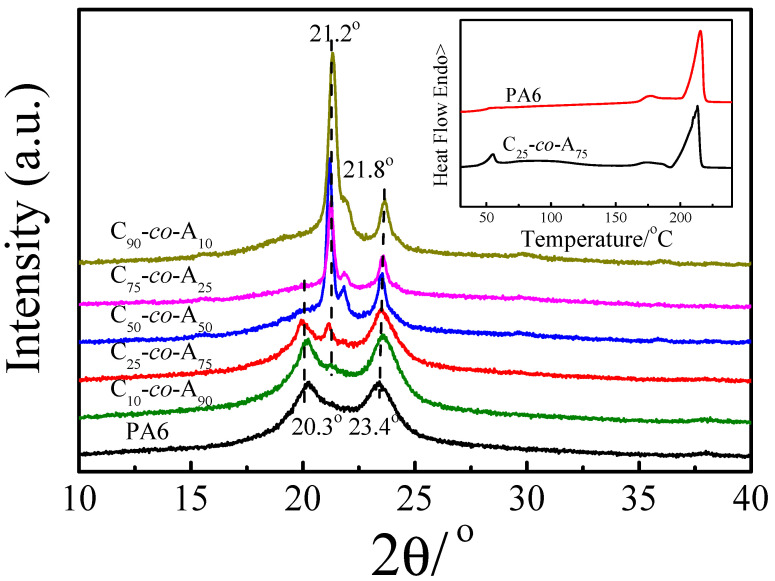
Wide-angle X-ray diffraction (WAXD) patterns of PA6 and P(CLA-*co*-CLO) copolymers after isothermal crystallization firstly at 160 °C for 2 h and then at 40 °C. The insert is the DSC melting curves of PA6 and C_25_*-co-*A_75_ with the same thermal history as the WAXD experiments. (The heating rate is 10 °C/min).

Figure 6 shows the WAXD patterns of PA6 and P(CLA-*co*-CLO) copolymers after isothermal crystallization first at 160 °C and then at 40 °C. The two diffraction peaks at 2θ = 20.3° and 23.4° correspond to the α-monoclinic crystal with the reflection planes (200) and (002) of PA6, respectively. The diffraction peaks located at 2θ = 21.2°, 22.0°, and 23.4° correspond to the (110), (111), and (200) reflections of PCL, respectively [22]. With the increase of CLO content, the diffraction peak at 2θ = 21.2° gradually appears and strengthen. It is reported that PA6 is a polycrystalline polymer, except for the α form, the unstable structure, which can form at lower crystallization temperature or rapid cooling process, is characterized by an intense peak at 2θ = 21° and a less intense one at 2θ = 11° [23]. In order to determine the attribution of the diffraction peak at 2θ = 21.2°, the DSC test on the sample with the same thermal history as WAXD was carried out, as shown inside Figure 6. The endothermic peak at 51.7 °C for C_25_*-co-*A_75_ suggest that PCL block crystallized. Also, the double melting peaks are similar to that of neat PA6. So the diffraction peak at 2θ = 21.2° should assign to the PCL crystals, and the diffraction peak at 2θ = 23.4° overlapped with the diffraction peak of PA6. As for C_50_*-co-*A_50_, the diffraction peaks at 21.2° and 23.4° are shaper and stronger, but the peak at 20.3° is weak, suggesting a large amount of PCL crystals and a small amount of PA6 crystals co-exist [12], which is consistent with the DSC results. Hence, it is concluded that the crystal structure of copolymer is influenced greatly by the content of CLO.

### 3.3. Water Absorption

**Figure 7:**
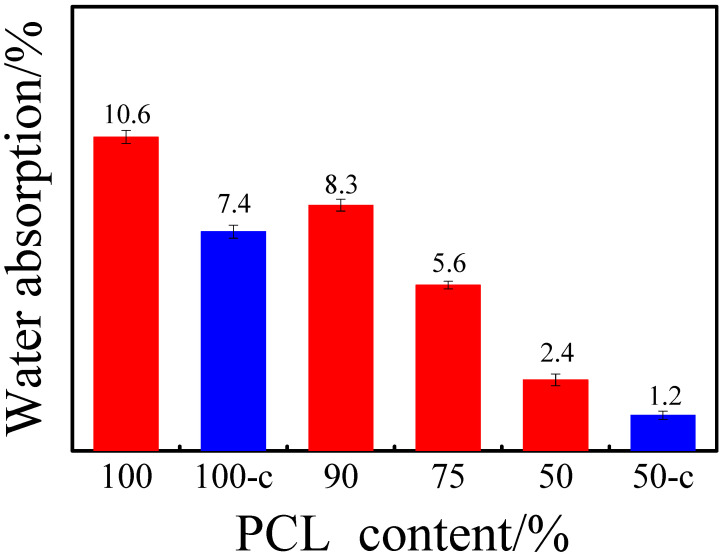
Water absorption of PA6 and P(CLA*-co-*CLO) copolymers. 100-c and 50-c are crystallized samples (firstly isothermally crystallized at 160 °C and then at 40 °C).

The intense polar amide groups in the PA6 molecule cause it to have strong water absorption. After water absorbing PA6 swells, its gas barrier, strength, and dimensional stability drop sharply. PCL is hydrophobic due to the ester bond, so the influence of PCL content on the water absorption of P(CLA-*co*-CLO) copolymers were summarized in Figure 7. It was found that the water absorption decreases with increasing PCL content for samples cooled down to room temperature naturally, which implies that the introduction of PCL is beneficial to reduce the water absorption of the copolymer. Moreover, the effect of crystallinity on the water absorption of P(CLA*-co-*CLO) copolymers is also examined. The water absorption of both PA6 and C_50_*-co-*A_50_ decrease greatly after crystallization [24]. The above results indicate that the water absorption of PA6 can be regulated by adjusting the crystallinity and the introduction content of hydrophobic polymers. The results of this work provide an option for the reduction of PA water absorption.

### 3.4. Biodegradation Behavior

As is known, PCL can be degraded by Lipase PS, which attacks the ester links along the chain. Meanwhile, PA6 is nonbiodegradable in most cases, which could be ascribed to strong hydrogen bonds and stable crystal structure as reported [8]. Many publications have confirmed that P(CLA-*co*-CLO) copolymers are biodegradable because of the presence of CLO units. Thus, the degradation behavior of P(CLA-*co*-CLO) copolymers was investigated.

**Figure 8:**
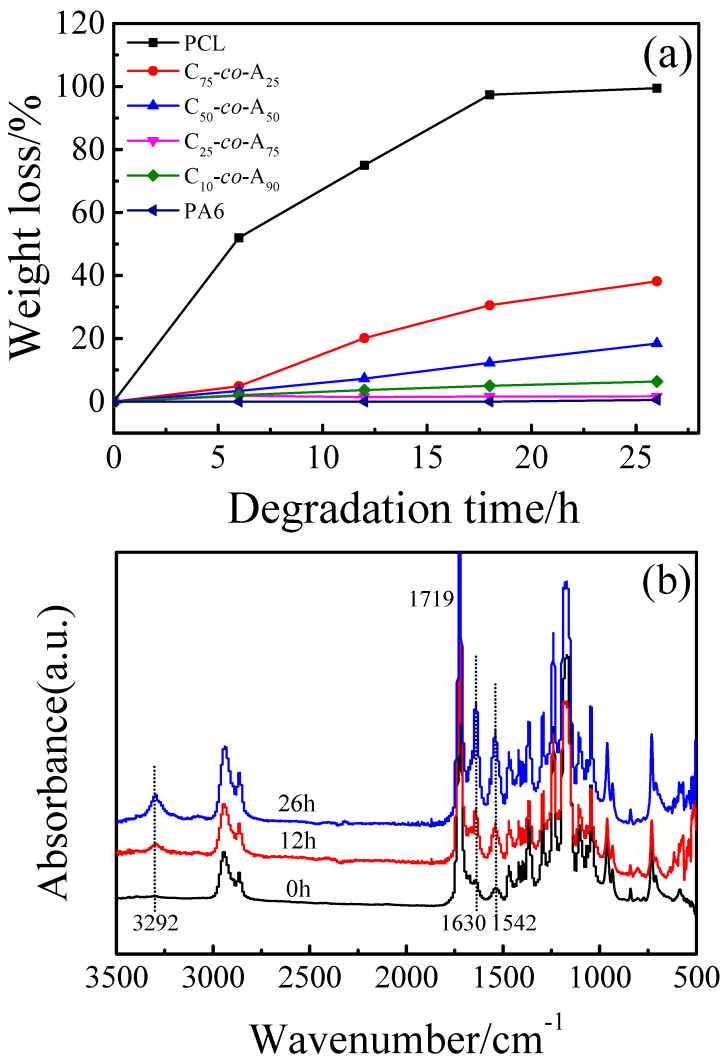
(**a**) Weight loss of PA6 and P(CLA-co-CLO) copolymers in the course of enzymatic degradation, and (**b**) normalized FTIR spectra of C_75_-*co*-A_25_ after degradation for different time.

Figure 8a presents the plots of weight loss versus degradation time of commercial PCL, PA6, and P(CLA-*co*-CLO) copolymers. It is clearly found that PA6 cannot be degraded by Lipase PS. For P(CLA-*co*-CLO) copolymers, the degradation rate increases as the PCL content increases, but the weight loss of copolymers is much lower than that of commercial PCL. After 26 h degradation, the weight loss of P(CLA-*co*-CLO) copolymers are lower than the calculated content of PCL by ^1^H NMR, indicating that the PCL blocks were not degraded completely. This may be due to PA6 blocks blocking the contact of PCL blocks with enzymes. The results of this work are completely different from the previous results of degradation in soil by Michell et al. [5]. They found that the random copolymers degraded much faster than PCL homopolymer and the total degradation (100% weight loss) was obtained for C_55_-*ran*-A_45_ and C_45_-*ran*-A_55_ copolymers. They attribute the above results to the fact that the CLO sequence in the random copolymer is mainly amorphous, because the neat PCL homopolymer used for comparison has a higher molecular weight and 43% crystallinity. It is difficult to compare the two works and draw accurate conclusions, because copolymers, homopolymers, and their preparation methods and degradation conditions are different. Fortunately, through the comparison of DSC and NMR results of C_25_-*co*-A_75_ copolymer in this work and C_55_-*ran*-A_45_ copolymer in Michell’s work, it is found that the chemical composition (the calculated CLO:CLA value of copolymers according to the ^1^H NMR results in this work is much higher, for C_75_-*co*-A_25_, it is 1.56) and the crystallization ability of CLO component (no crystallization peak was found for the CLO segment during the cooling process, but a small melting peak appeared during the heating process) is similar, but the ^13^C NMR results suggest that the copolymers synthesized by Michell et al. have a higher degree of randomness. meanwhile, the homopolymers used in the two works for degradation comparison are all neat PCL with higher molecular weight. Therefore, the different degradation results maybe have resulted from the different randomness of copolymers, and the higher randomness may be conducive to the degradation of the copolymers. The break of the CLO ester bond at both ends of the CLA sequence in P(CLA-*co*-CLO) copolymers with higher randomness will cause CLA fragments spread into the soil, which can also explain why the total degradation can be achieved for C_55_-*ran*-A_45_ and the C_45_-*ran*-A_55_ copolymers.

In order to explain the changes before and after degradation, the normalized FTIR spectra of C**_75_**-***co***-A**_25_** are shown in Figure 8b. For comparison, the spectra were normalized to the maximum of *v*(C=O) vibration at 1719 cm^−1^ which is assigned to the CLO segments. The peak intensity at 3292, 1630, and 1542 cm^−1^ gradually increased with the increase of degradation time, indicating that the PA6 content increased as degradation time increase, which is consistent with the assumption that the degradation is mainly derived from PCL blocks.

## 4. Conclusions

A series of P(CLA-*co*-CLO) copolymers were successfully synthesized by anionic copolymerization, and the composition was regulated by the molar feed ratio. The crystallization behaviour of P(CLA-*co*-CLO) copolymers was affected by the composition of the copolymer. When the designed content of the two components are all higher than 25%, both PCL and PA6 blocks can crystallize. The PCL block decreased the crystallization rate of PA6 block but had little effect on the melting behavior of PA6. While the crystallized PA6 acted as heterogeneous nucleating agent and greatly improved the crystallization rate of PCL block. Moreover, the introduction of PCL greatly reduced the water absorption of P(CLA-*co*-CLO) copolymers and endowed them a certain degree of degradability. The results of this work can provide a simple method for adjusting the properties of poly(ester amide)s.

## Figures and Tables

**Table 1:** The molar ratio and differential scanning calorimetry (DSC) data of P(CLA*-co-*CLO) copolymers.

Sample	CLO:CLA		Cooling Scan				Second Heating			
	Feed Ratio	M_PCLO_:M_PCLA_^a^	*T*_cc_ (°C)		Δ*H*_c_ (J/g)		*T*_m_ (°C)		Δ*H*_m_ (J/g)	
			PCL	PA6	PCL	PA6	PCL	PA6	PCL	PA6
PA6	0:100	-	-	173.8	-	66.4	-	212.7	-	54.2
C_10_*-co-*A_90_	10:90	18:82	-	167.2	-	59.6	-	213.4	-	46.3
C_25_*-co-*A_75_	25:75	61:39	-	168.7	-	52.9	-	212.8	-	49.5
C_50_*-co-*A_50_	50:50	76:24	27.0	142.2	36.2	9.0	56.8	201.4	39.9	10.6
C_75_*-co-*A_25_	75:25	80:20	26.8	139.4	57.4	6.6	59.2	191.9	54.2	5.9
C_75_*-co-*A_25_-a	75:25	-	25.3	-	64.9	-	53.6	-	70.9	-
C_90_*-co-*A_10_	90:10	-	25.3	-	70.9		53.4	-	78.3	

^a^ The molar ratio determined by ^1^H NMR.

## References

[1] Becker G., Wurm F.R. (2018). Functional biodegradable polymers via ring-opening polymerization of monomers without protective groups. Chem. Soc. Rev..

[2] Torres E., Dominguez-Candela I., Castello-Palacios S., Vallés-Lluch A., Fombuena V. (2020). Development and characterization of polyester and acrylate-based composites with hydroxyapatite and halloysite nanotubes for medical applications. Polymers.

[3] Duan X., Wu Y., Chen Z., Yang T., Cheng Y., Yu H., Huang T. (2019). In-situ polymerization of high-molecular weight nylon 66 modified clay nanocomposites with low apparent viscosity. Polymers.

[4] Huang C.N., Wu C.M., Lo H.W., Lai C.C., Teng W.F., Liu L.C., Chen C.M. (2019). Synthesis and physical properties of non-crystalline nylon 6 containing dimer acid. Polymers.

[5] Michell R.M., Müller A.J., Castelletto V., Hamley I., Deshayes G.L., Dubois P. (2009). Effect of sequence distribution on the morphology, crystallization, melting, and biodegradation of poly(ε-caprolactone-co-ε-caprolactam) copolymers. Macromolecules.

[6] Walha F., Lamnawar K., Maazouz A., Jaziri M. (2016). Rheological, morphological and mechanical studies of sustainably sourced polymer blends based on poly(lactic acid) and polyamide 11. Polymers.

[7] Goodman R.N.V. (1984). Copolyesteramides—II anionic copolymers of ε-caprolactam with ε-caprolactone. Macromolecules.

[8] Gonsalves K.E., Chen X., Cameron J.A. (1992). Degradation of nonalternating poly(ester-amides). Macromolecules.

[9] Deshayes G., Delcourt C., Verbruggen I., Trouillet-Fonti L., Touraud F., Fleury E., Degée P., Destarac M., Willem R., Dubois P. (2009). Novel polyesteramide-based diblock copolymers: Synthesis by ring-opening copolymerization and characterization. Macromol. Chem. Phys..

[10] Liu S.Y., Li C.G., Zhao J.B., Zhang Z.Y., Yang W.T. (2011). Synthesis and characterization of polyesteramides having short nylon-6 segments. Polymer.

[11] Sanchez-Sanchez A., Basterretxea A., Mantione D., Etxeberria A., Elizetxea C., Calle A., García-Arrieta S., Sardon H., Mecerreyes D. (2016). Organic-acid mediated bulk polymerization of ε-caprolactam and its copolymerization with ε-caprolactone. J. Polym. Sci. Part A Polym. Chem..

[12] Zeng F.R., Xu J., Sun L.H., Ma J., Jiang H., Li Z.L. (2020). Copolymers of ε-caprolactone and ε-caprolactam via polyesterification: Towards sequence-controlled poly(ester amide)s. Polym. Chem..

[13] Zhang S., Zhang J., Tang L., Huang J., Fang Y., Ji P., Wang C., Wang H. (2019). A novel synthetic strategy for preparing polyamide 6 (PA6)-based polymer with transesterification. Polymers.

[14] Cakir S., Kierkels R., Koning C. (2011). Polyamide 6-polycaprolactone multiblock copolymers: Synthesis, characterization, and degradation. J. Polym. Sci. Part A Polym. Chem..

[15] Michell R.M., Müller A.J., Deshayes G., Dubois P. (2010). Effect of sequence distribution on the isothermal crystallization kinetics and successive self-nucleation and annealing (SSA) behavior of poly(ε-caprolactone-co-ε-caprolactam) copolymers. Eur. Polym. J..

[16] Deshayes G., Delcourt C., Verbruggen I., Trouillet-Fonti L., Touraud F., Fleury E., Degée P., Destarac M., Willem R., Dubois P. (2008). Activation of the hydrolytic polymerization of ε-caprolactam by ester functions: Straightforward route to aliphatic polyesteramides. React. Funct. Polym..

[17] Deshayes G., Delcourt C., Verbruggen I., Trouillet-Fonti L., Touraud F., Fleury E., Degée P., Destarac M., Willem R., Dubois P. (2011). Novel polyesteramide-based di- and triblock copolymers: From thermo-mechanical properties to hydrolytic degradation. Eur. Polym. J..

[18] Chen X., Gonsalves K.E., Cameron J.A. (1993). Further studies on biodegradation of aliphatic poly (ester-amides). J. Appl. Polym. Sci..

[19] Rusu E., Rusu G., Rusu D. (2019). Effects of temperature and comonomer content on poly(ε-caprolactam-co-ε-caprolactone) copolymers properties: An evaluation of structural changes and dielectric behavior. Polym. Eng. Sci..

[20] Xue F., Jiang S. (2014). Crystallization behaviors and structure transitions of biocompatible and biodegradable diblock copolymers. Polymers.

[21] Eastmond G.C., Eastmond G.C., Höcker H., Klee D. (1999). Poly(ε-caprolactone) blends. Biomedical Applications Polymer Blends.

[22] Sun Z., Choi B., Feng A., Moad G., Thang S.H. (2019). Nonmigratory poly(vinyl chloride)-block-polycaprolactone plasticizers and compatibilizers prepared by sequential RAFT and ring-opening polymerization (RAFT-T-ROP). Macromolecules.

[23] Newman D., Laredo E., Bello A., Dubois P. (2014). Combined effect of humidity and composition on the molecular mobilities of poly(ε-caprolactone-ran-ε-caprolactam) copolymers. Macromolecules.

[24] Heidarzadeh N., Rafizadeh M., Taromi F.A., Puiggalí J., Del Valle L.J., Franco L. (2017). Preparation of random poly(butylene alkylate-co-terephthalate)s with different methylene group contents: Crystallization and degradation kinetics. J. Polym. Res..

